# Ethyl 1-(2-hy­droxy­eth­yl)-2-[2-(methyl­sulfan­yl)eth­yl]-1*H*-benzimidazole-5-carboxyl­ate

**DOI:** 10.1107/S160053681105389X

**Published:** 2011-12-21

**Authors:** Nurasyikin Hamzah, Nurziana Ngah, Shafida Abd Hamid, Aisyah Saad Abdul Rahim

**Affiliations:** a Kulliyyah of Science, International Islamic University Malaysia, Bandar Indera Mahkota, 25200 Kuantan, Pahang, Malaysia; b School of Pharmaceutical Sciences, Universiti Sains Malaysia, 11800 Penang, Malaysia

## Abstract

In the crystal structure of the title compound, C_15_H_20_N_2_O_3_S, the hy­droxy group is involved in the formation of O—H⋯N hydrogen bonds, which link two mol­ecules into a centrosymmetric dimer. Weak C—H⋯O hydrogen bonds further link these dimers into chains propagating along the *a* axis. The crystal packing exhibits π–π inter­actions between the five- and six-membered rings of neighbouring mol­ecules [centroid–centroid distance = 3.819 (2) Å] and short inter­molecular S⋯S contacts of 3.495 (1) Å.

## Related literature

For details of the synthesis and related structures, see: Wright (1951 ▶); Preston (1974 ▶); Hamzah *et al.* (2010 ▶); Arumugam *et al.* 2011 ▶); Ruiz *et al.* (2010 ▶); Chou *et al.* (2011 ▶). For the therapeutic properties of benzimidazole derivatives, see: Li *et al.* (2006 ▶); Hwu *et al.* (2008 ▶); Cui *et al.* (2010 ▶); Sasmal *et al.* (2011 ▶); Demirayak *et al.* (2011 ▶). For bond lengths in organic compounds, see: Allen *et al.* (1987 ▶). For the low-temperature device used in the data collection, see: Cosier & Glazer (1986 ▶).
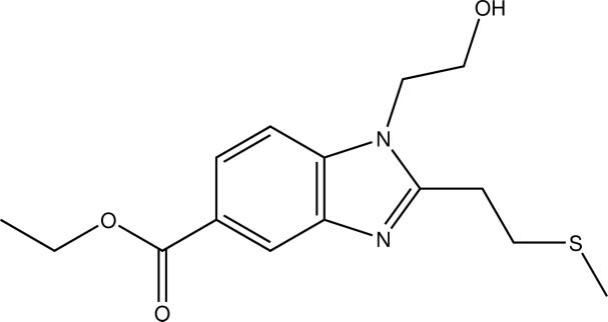


## Experimental

### 

#### Crystal data

C_15_H_20_N_2_O_3_S
*M* *_r_* = 308.39
Triclinic, 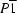

*a* = 8.3909 (1) Å
*b* = 8.8277 (2) Å
*c* = 11.5025 (2) Å
α = 110.218 (1)°
β = 102.529 (1)°
γ = 99.101 (1)°
*V* = 754.78 (2) Å^3^
*Z* = 2
Mo *K*α radiation
μ = 0.23 mm^−1^
*T* = 100 K
0.27 × 0.24 × 0.07 mm

#### Data collection

Bruker SMART APEXII CCD area-detector diffractometer
Absorption correction: multi-scan (*SADABS*; Bruker, 2009 ▶) *T*_min_ = 0.941, *T*_max_ = 0.985
6027 measured reflections
2627 independent reflections
2243 reflections with *I* > 2σ(*I*)
*R*_int_ = 0.026

#### Refinement

*R*[*F* ^2^ > 2σ(*F* ^2^)] = 0.037
*wR*(*F* ^2^) = 0.086
*S* = 1.04
2627 reflections
196 parameters
1 restraint
H atoms treated by a mixture of independent and constrained refinement
Δρ_max_ = 0.24 e Å^−3^
Δρ_min_ = −0.26 e Å^−3^

### 

Data collection: *APEX2* (Bruker, 2009 ▶); cell refinement: *SAINT* (Bruker, 2009 ▶); data reduction: *SAINT*; program(s) used to solve structure: *SHELXTL* (Sheldrick, 2008 ▶); program(s) used to refine structure: *SHELXTL*; molecular graphics: *SHELXTL*; software used to prepare material for publication: *SHELXTL* and *PLATON* (Spek, 2009 ▶).

## Supplementary Material

Crystal structure: contains datablock(s) global, I. DOI: 10.1107/S160053681105389X/cv5217sup1.cif

Structure factors: contains datablock(s) I. DOI: 10.1107/S160053681105389X/cv5217Isup2.hkl

Supplementary material file. DOI: 10.1107/S160053681105389X/cv5217Isup3.cml

Additional supplementary materials:  crystallographic information; 3D view; checkCIF report

## Figures and Tables

**Table 1:** Hydrogen-bond geometry (Å, °)

*D*—H⋯*A*	*D*—H	H⋯*A*	*D*⋯*A*	*D*—H⋯*A*
O3—H3⋯N1^i^	0.84 (2)	2.01 (2)	2.808 (2)	159 (2)
C11—H11*A*⋯O1^ii^	0.99	2.39	3.224 (2)	142
C11—H11*B*⋯O3^iii^	0.99	2.42	3.222 (2)	138

Symmetry codes: (i) 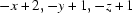; (ii) 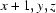; (iii) 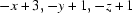.

**Fig. 1.**
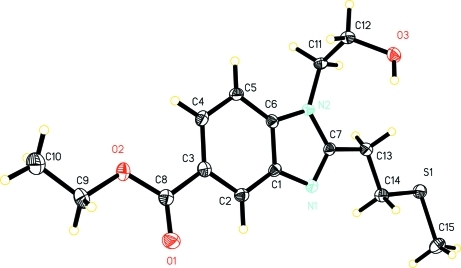
The molecular structure of (I) showing the atomic numbering and 50% probability displacement ellipsods.

**Fig. 2.**
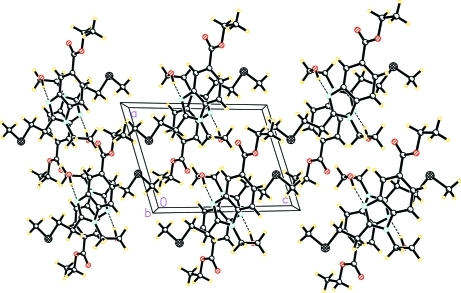
A portion of the crystal packing viewed down the b axis and showing the hydrogen-bonded (dashed lines) dimers.

## supplementary crystallographic information

### Comment

Heteroaromatic compounds of benzimidazoles exhibit important values particularly in biological and pharmaceutical fields. The previledge sub-structures of benzimidazoles have been reported as potential inhibitors (Sasmal *et al.*, 2011), probes for β-amyloid (Aβ) plaques in Alzheimer's disease (Cui *et al.*, 2010), showed anti- cancer activities (Demirayak *et al.*, 2011), anti hepatitis B (Li *et al.*, 2006) and C virus (Hwu *et al.*, 2008). Various methods have been employed to synthesize benzimidazole derivatives (Wright, 1951; Preston, 1974). However, two common methods widely used are either by reacting diamine with carboxylic acid or diamine with aldehyde using solid catalyst (Ruiz *et al.*, 2010) or polymer bounds (Chou *et al.*, 2011). In continuation of our work (Hamzah *et al.*, 2010; Arumugam *et al.* 2011), we report herein the crystal structure of the title compound, (I).

The title molecule (Fig. 1), is similar with those reported earlier - ethyl 1-(2- hydroxyethyl)-2-phenyl-1*H*-benzimidazole-5-carboxylate (Hamzah *et al.*, 2010) and ethyl 1-(2-hydroxyethyl)-2-propyl-1*H*-benzimidazole -5-carboxylate (Hamzah *et al.*, 2011), in that only the 2-methylthioethyl substituent at the imidazole ring is different. The benzimidazole ring [N1/N2/C1—C7] is essentially planar and the C4 atom deviates by 0.012 (2)Å from that plane. The bond lengths and angles are in normal ranges (Allen *et al.*, 1987) and are in agreement with those reported by Hamzah *et al.* (2010, 2011).

In the crystal structure, the hydroxy group is involved in formation intermolecular hydrogen bond O—H···N (Table 1), which link two molecules into centrosymmetric dimer  (Fig. 2). Weak intermolecular C—H···O hydrogen bonds (Table 1) link further these dimers into chains propagated along *a* axis. The crystal packing exhibits π–π interactions between the benzimidazole fragments with *Cg*1···*Cg*2 distance of  3.819 (2)Å (*Cg*1 and *Cg*2 are centroids of N1/N2/C1/C6/C7 and C1-C6, respectively) and short intermolecular S···S contacts of 3.495 (1) Å.

### Experimental

A mixture of 3-amino-4-(2-hydroxylethanolamine)benzoic acid ethyl ester (0.1.0 g, 0.45 mmol), K10-montmorillonite (2.0 g), 3-(methylthio) propionaldehyde (0.098 g, 0.91 mmol) and 1 ml MeCN were irradiated in CEM^TM^ microwave at 80°C, 150 W, 5 bar and hold for 5 minutes. Then, another aliquot of aldehyde was added and the reation mixture was irradiated again at the same condition as before. The reaction was monitored by TLC (Hex:EtOAc,1:4). Upon completion, K10-montmorillonite was removed by filtration, washed with DCM and later evaporated *in vacuo* to afford brown precipitate. The compound was purified with PLC (Hex:EtOAc, 1:4) before it was recrystallized with hot MeOH to afford colourless crystals.

### Refinement

X-ray data were collected at 100 K (Cosier & Glazer, 1986). Hydroxyl atom H3 was located from difference Fourier map, and isotropically refined with restraint O3—H3 = 0.843 (10) Å. The remaining H atoms attached to C atoms were fixed geometrically and refined as riding, with C—H= 0.95–0.99Å and with *U*_iso_(H)=1.2 or 1.5*U*_eq_(C). A rotating group model was applied to the methyl groups.

### Crystal data

**Table:**

C_15_H_20_N_2_O_3_S	*Z* = 2
*M**_r_* = 308.39	*F*(000) = 328
Triclinic, *P*1	*D*_x_ = 1.357 Mg m^−^^3^
Hall symbol: -P 1	Mo *K*α radiation, λ = 0.71073 Å
*a* = 8.3909 (1) Å	Cell parameters from 2401 reflections
*b* = 8.8277 (2) Å	θ = 1.9–25.0°
*c* = 11.5025 (2) Å	µ = 0.23 mm^−^^1^
α = 110.218 (1)°	*T* = 100 K
β = 102.529 (1)°	Plate, colourless
γ = 99.101 (1)°	0.27 × 0.24 × 0.07 mm
*V* = 754.78 (2) Å^3^	

### Data collection

**Table:**

Bruker SMART APEXII CCD area-detector diffractometer	2627 independent reflections
Radiation source: fine-focus sealed tube	2243 reflections with *I* > 2σ(*I*)
graphite	*R*_int_ = 0.026
Detector resolution: 83.66 pixels mm^-1^	θ_max_ = 25.0°, θ_min_ = 1.9°
φ and ω scan	*h* = −9→9
Absorption correction: multi-scan (*SADABS*; Bruker, 2009)	*k* = −9→10
*T*_min_ = 0.941, *T*_max_ = 0.985	*l* = −13→13
6027 measured reflections	

### Refinement

**Table:**

Refinement on *F*^2^	Primary atom site location: structure-invariant direct methods
Least-squares matrix: full	Secondary atom site location: difference Fourier map
*R*[*F*^2^ > 2σ(*F*^2^)] = 0.037	Hydrogen site location: inferred from neighbouring sites
*wR*(*F*^2^) = 0.086	H atoms treated by a mixture of independent and constrained refinement
*S* = 1.04	*w* = 1/[σ^2^(*F*_o_^2^) + (0.0307*P*)^2^ + 0.5234*P*] where *P* = (*F*_o_^2^ + 2*F*_c_^2^)/3
2627 reflections	(Δ/σ)_max_ < 0.001
196 parameters	Δρ_max_ = 0.24 e Å^−^^3^
1 restraint	Δρ_min_ = −0.26 e Å^−^^3^

### Special details

**Table:**

Experimental. The crystal was placed in the cold stream of an Oxford Cryosystems Cobra open=flow nitrogen cryostat (Cosier & Glazer, 1986) operating at 100.0 (1) K.
Geometry. All e.s.d.'s (except the e.s.d. in the dihedral angle between two l.s. planes) are estimated using the full covariance matrix. The cell e.s.d.'s are taken into account individually in the estimation of e.s.d.'s in distances, angles and torsion angles; correlations between e.s.d.'s in cell parameters are only used when they are defined by crystal symmetry. An approximate (isotropic) treatment of cell e.s.d.'s is used for estimating e.s.d.'s involving l.s. planes.
Refinement. Refinement of *F*^2^ against ALL reflections. The weighted *R*-factor *wR* and goodness of fit *S* are based on *F*^2^, conventional *R*-factors *R* are based on *F*, with *F* set to zero for negative *F*^2^. The threshold expression of *F*^2^ > σ(*F*^2^) is used only for calculating *R*-factors(gt) *etc*. and is not relevant to the choice of reflections for refinement. *R*-factors based on *F*^2^ are statistically about twice as large as those based on *F*, and *R*- factors based on ALL data will be even larger.

### Fractional atomic coordinates and isotropic or equivalent isotropic displacement parameters (Å^2^)

**Table:**

	*x*	*y*	*z*	*U*_iso_*/*U*_eq_	
S1	1.33465 (6)	0.81922 (6)	0.91756 (5)	0.02048 (15)	
O1	0.36330 (17)	−0.07772 (17)	0.28863 (14)	0.0244 (3)	
O2	0.46252 (16)	−0.29862 (16)	0.19992 (13)	0.0195 (3)	
O3	1.32576 (17)	0.48466 (17)	0.39325 (14)	0.0213 (3)	
H3	1.2305 (18)	0.505 (3)	0.391 (2)	0.035 (7)*	
N1	0.94054 (19)	0.36073 (19)	0.58614 (15)	0.0151 (3)	
N2	1.14023 (19)	0.26662 (19)	0.50164 (15)	0.0140 (3)	
C1	0.8668 (2)	0.2070 (2)	0.48209 (18)	0.0140 (4)	
C2	0.6994 (2)	0.1144 (2)	0.43009 (18)	0.0158 (4)	
H2	0.6138	0.1549	0.4640	0.019*	
C3	0.6610 (2)	−0.0396 (2)	0.32689 (18)	0.0153 (4)	
C4	0.7890 (2)	−0.0998 (2)	0.27731 (18)	0.0166 (4)	
H4	0.7597	−0.2063	0.2081	0.020*	
C5	0.9553 (2)	−0.0084 (2)	0.32637 (18)	0.0164 (4)	
H5	1.0408	−0.0485	0.2921	0.020*	
C6	0.9912 (2)	0.1465 (2)	0.42935 (18)	0.0141 (4)	
C7	1.1022 (2)	0.3904 (2)	0.59415 (18)	0.0145 (4)	
C8	0.4814 (2)	−0.1364 (2)	0.27111 (18)	0.0169 (4)	
C9	0.2882 (3)	−0.3974 (2)	0.1397 (2)	0.0231 (5)	
H9A	0.2271	−0.3537	0.0798	0.028*	
H9B	0.2301	−0.3927	0.2067	0.028*	
C10	0.2919 (3)	−0.5719 (3)	0.0675 (2)	0.0391 (6)	
H10A	0.3534	−0.5743	0.0038	0.059*	
H10B	0.1762	−0.6412	0.0227	0.059*	
H10C	0.3486	−0.6153	0.1282	0.059*	
C11	1.3032 (2)	0.2629 (2)	0.47536 (19)	0.0165 (4)	
H11A	1.3195	0.1490	0.4552	0.020*	
H11B	1.3949	0.3403	0.5538	0.020*	
C12	1.3139 (2)	0.3126 (2)	0.36286 (19)	0.0181 (4)	
H12A	1.4139	0.2852	0.3363	0.022*	
H12B	1.2125	0.2463	0.2884	0.022*	
C13	1.2355 (2)	0.5380 (2)	0.69567 (18)	0.0171 (4)	
H13A	1.2923	0.6013	0.6533	0.021*	
H13B	1.3214	0.4989	0.7436	0.021*	
C14	1.1634 (2)	0.6532 (2)	0.79092 (18)	0.0179 (4)	
H14A	1.0995	0.5893	0.8293	0.022*	
H14B	1.0850	0.7012	0.7452	0.022*	
C15	1.2132 (3)	0.9482 (3)	0.9979 (2)	0.0253 (5)	
H15A	1.1466	0.9862	0.9373	0.038*	
H15B	1.2896	1.0452	1.0725	0.038*	
H15C	1.1373	0.8836	1.0275	0.038*	

### Atomic displacement parameters (Å^2^)

**Table:**

	*U*^11^	*U*^22^	*U*^33^	*U*^12^	*U*^13^	*U*^23^
S1	0.0191 (3)	0.0174 (3)	0.0193 (3)	0.0018 (2)	0.0047 (2)	0.0024 (2)
O1	0.0171 (8)	0.0243 (8)	0.0270 (8)	0.0040 (6)	0.0062 (6)	0.0053 (7)
O2	0.0192 (7)	0.0149 (7)	0.0191 (7)	0.0003 (6)	0.0016 (6)	0.0046 (6)
O3	0.0165 (8)	0.0198 (7)	0.0332 (9)	0.0064 (6)	0.0106 (7)	0.0144 (7)
N1	0.0152 (8)	0.0137 (8)	0.0161 (8)	0.0033 (6)	0.0047 (7)	0.0054 (7)
N2	0.0126 (8)	0.0142 (8)	0.0167 (8)	0.0043 (6)	0.0057 (7)	0.0065 (7)
C1	0.0168 (10)	0.0131 (9)	0.0135 (10)	0.0056 (8)	0.0046 (8)	0.0062 (8)
C2	0.0161 (10)	0.0172 (10)	0.0160 (10)	0.0059 (8)	0.0058 (8)	0.0075 (8)
C3	0.0174 (10)	0.0162 (10)	0.0144 (10)	0.0040 (8)	0.0038 (8)	0.0090 (8)
C4	0.0224 (11)	0.0129 (9)	0.0141 (10)	0.0039 (8)	0.0054 (8)	0.0049 (8)
C5	0.0189 (10)	0.0167 (10)	0.0175 (10)	0.0070 (8)	0.0095 (8)	0.0077 (8)
C6	0.0143 (10)	0.0146 (9)	0.0153 (10)	0.0037 (8)	0.0043 (8)	0.0083 (8)
C7	0.0176 (10)	0.0132 (9)	0.0153 (10)	0.0057 (8)	0.0059 (8)	0.0074 (8)
C8	0.0208 (11)	0.0174 (10)	0.0136 (10)	0.0040 (8)	0.0049 (8)	0.0079 (8)
C9	0.0184 (11)	0.0230 (11)	0.0224 (11)	−0.0025 (8)	0.0015 (9)	0.0085 (9)
C10	0.0296 (13)	0.0301 (13)	0.0410 (15)	−0.0038 (10)	0.0105 (12)	−0.0004 (11)
C11	0.0140 (10)	0.0162 (10)	0.0213 (11)	0.0070 (8)	0.0075 (8)	0.0070 (8)
C12	0.0170 (10)	0.0185 (10)	0.0219 (11)	0.0072 (8)	0.0094 (9)	0.0082 (9)
C13	0.0161 (10)	0.0169 (10)	0.0183 (11)	0.0038 (8)	0.0056 (8)	0.0065 (8)
C14	0.0161 (10)	0.0187 (10)	0.0161 (10)	0.0032 (8)	0.0034 (8)	0.0047 (8)
C15	0.0294 (12)	0.0214 (11)	0.0222 (11)	0.0087 (9)	0.0075 (10)	0.0043 (9)

### Geometric parameters (Å, °)

**Table:**

S1—C15	1.799 (2)	C5—H5	0.9500
S1—C14	1.812 (2)	C7—C13	1.495 (3)
O1—C8	1.212 (2)	C9—C10	1.485 (3)
O2—C8	1.345 (2)	C9—H9A	0.9900
O2—C9	1.456 (2)	C9—H9B	0.9900
O3—C12	1.417 (2)	C10—H10A	0.9800
O3—H3	0.843 (10)	C10—H10B	0.9800
N1—C7	1.317 (2)	C10—H10C	0.9800
N1—C1	1.395 (2)	C11—C12	1.518 (3)
N2—C7	1.372 (2)	C11—H11A	0.9900
N2—C6	1.379 (2)	C11—H11B	0.9900
N2—C11	1.466 (2)	C12—H12A	0.9900
C1—C2	1.391 (3)	C12—H12B	0.9900
C1—C6	1.403 (2)	C13—C14	1.521 (3)
C2—C3	1.393 (3)	C13—H13A	0.9900
C2—H2	0.9500	C13—H13B	0.9900
C3—C4	1.414 (3)	C14—H14A	0.9900
C3—C8	1.485 (3)	C14—H14B	0.9900
C4—C5	1.379 (3)	C15—H15A	0.9800
C4—H4	0.9500	C15—H15B	0.9800
C5—C6	1.400 (3)	C15—H15C	0.9800
			
C15—S1—C14	99.19 (10)	C9—C10—H10A	109.5
C8—O2—C9	114.83 (15)	C9—C10—H10B	109.5
C12—O3—H3	110.8 (17)	H10A—C10—H10B	109.5
C7—N1—C1	104.86 (15)	C9—C10—H10C	109.5
C7—N2—C6	106.81 (14)	H10A—C10—H10C	109.5
C7—N2—C11	128.13 (16)	H10B—C10—H10C	109.5
C6—N2—C11	124.94 (15)	N2—C11—C12	111.81 (15)
C2—C1—N1	130.07 (17)	N2—C11—H11A	109.3
C2—C1—C6	120.17 (17)	C12—C11—H11A	109.3
N1—C1—C6	109.76 (16)	N2—C11—H11B	109.3
C1—C2—C3	118.05 (17)	C12—C11—H11B	109.3
C1—C2—H2	121.0	H11A—C11—H11B	107.9
C3—C2—H2	121.0	O3—C12—C11	113.02 (16)
C2—C3—C4	120.76 (17)	O3—C12—H12A	109.0
C2—C3—C8	117.54 (17)	C11—C12—H12A	109.0
C4—C3—C8	121.69 (17)	O3—C12—H12B	109.0
C5—C4—C3	121.96 (18)	C11—C12—H12B	109.0
C5—C4—H4	119.0	H12A—C12—H12B	107.8
C3—C4—H4	119.0	C7—C13—C14	112.12 (15)
C4—C5—C6	116.42 (17)	C7—C13—H13A	109.2
C4—C5—H5	121.8	C14—C13—H13A	109.2
C6—C5—H5	121.8	C7—C13—H13B	109.2
N2—C6—C5	131.88 (17)	C14—C13—H13B	109.2
N2—C6—C1	105.50 (16)	H13A—C13—H13B	107.9
C5—C6—C1	122.61 (17)	C13—C14—S1	109.23 (13)
N1—C7—N2	113.06 (17)	C13—C14—H14A	109.8
N1—C7—C13	124.93 (16)	S1—C14—H14A	109.8
N2—C7—C13	121.95 (16)	C13—C14—H14B	109.8
O1—C8—O2	122.83 (18)	S1—C14—H14B	109.8
O1—C8—C3	124.28 (18)	H14A—C14—H14B	108.3
O2—C8—C3	112.88 (16)	S1—C15—H15A	109.5
O2—C9—C10	107.31 (17)	S1—C15—H15B	109.5
O2—C9—H9A	110.3	H15A—C15—H15B	109.5
C10—C9—H9A	110.3	S1—C15—H15C	109.5
O2—C9—H9B	110.3	H15A—C15—H15C	109.5
C10—C9—H9B	110.3	H15B—C15—H15C	109.5
H9A—C9—H9B	108.5		
			
C7—N1—C1—C2	179.65 (19)	C1—N1—C7—C13	−177.00 (17)
C7—N1—C1—C6	0.33 (19)	C6—N2—C7—N1	−0.9 (2)
N1—C1—C2—C3	−178.17 (17)	C11—N2—C7—N1	175.16 (16)
C6—C1—C2—C3	1.1 (3)	C6—N2—C7—C13	176.55 (16)
C1—C2—C3—C4	0.4 (3)	C11—N2—C7—C13	−7.4 (3)
C1—C2—C3—C8	−179.23 (16)	C9—O2—C8—O1	3.0 (3)
C2—C3—C4—C5	−1.4 (3)	C9—O2—C8—C3	−178.05 (15)
C8—C3—C4—C5	178.19 (17)	C2—C3—C8—O1	16.9 (3)
C3—C4—C5—C6	0.8 (3)	C4—C3—C8—O1	−162.71 (18)
C7—N2—C6—C5	−177.37 (19)	C2—C3—C8—O2	−162.02 (16)
C11—N2—C6—C5	6.4 (3)	C4—C3—C8—O2	18.4 (2)
C7—N2—C6—C1	1.00 (19)	C8—O2—C9—C10	−179.17 (17)
C11—N2—C6—C1	−175.20 (16)	C7—N2—C11—C12	−98.5 (2)
C4—C5—C6—N2	178.81 (18)	C6—N2—C11—C12	76.8 (2)
C4—C5—C6—C1	0.7 (3)	N2—C11—C12—O3	70.8 (2)
C2—C1—C6—N2	179.76 (16)	N1—C7—C13—C14	0.0 (3)
N1—C1—C6—N2	−0.84 (19)	N2—C7—C13—C14	−177.14 (16)
C2—C1—C6—C5	−1.7 (3)	C7—C13—C14—S1	175.48 (13)
N1—C1—C6—C5	177.72 (16)	C15—S1—C14—C13	171.67 (14)
C1—N1—C7—N2	0.3 (2)		

### Hydrogen-bond geometry (Å, °)

**Table:**

*D*—H···*A*	*D*—H	H···*A*	*D*···*A*	*D*—H···*A*
O3—H3···N1^i^	0.84 (2)	2.01 (2)	2.808 (2)	159 (2)
C11—H11A···O1^ii^	0.99	2.39	3.224 (2)	142
C11—H11B···O3^iii^	0.99	2.42	3.222 (2)	138

Symmetry codes: (i) −*x*+2, −*y*+1, −*z*+1; (ii) *x*+1, *y*, *z*; (iii) −*x*+3, −*y*+1, −*z*+1.

## References

[bb1] Allen, F. H., Kennard, O., Watson, D. G., Brammer, L., Orpen, A. G. & Taylor, R. (1987). *J. Chem. Soc. Perkin Trans. 2*, pp. S1–19.

[bb8] Arumugam, N., Ngah, N., Abd Hamid, S. & Abdul Rahim, A. S. (2011). *Acta Cryst.* E**67**, o2938. 10.1107/S1600536811041663 PMC3247350 22219968

[bb2] Bruker (2009). *APEX2*, *SAINT* and *SADABS* Bruker AXS Inc., Madison, Wisconsin, USA.

[bb3] Chou, C.-T., Yellol, G. S., Chang, W.-J., Sun, M.-L. & Sun, C.-M. (2011). *Tetrahedron*, **67**, 2110–2117.

[bb4] Cosier, J. & Glazer, A. M. (1986). *J. Appl. Cryst.* **19**, 105–107.

[bb5] Cui, M., Ono, M., Kimura, H., Kawashima, H., Liu, B. L. & Saji, H. (2010). *Nucl. Med. Biol.* **38**, 313–320. 10.1016/j.nucmedbio.2010.09.012 21492779

[bb6] Demirayak, S., Kajagil, I. & Yurttas, L. (2011). *Eur. J. Med. Chem.* **46**, 411–416. 10.1016/j.ejmech.2010.11.007 21122952

[bb7] Hamzah, N., Abd. Hamid, S., Abdul Rahim, A. S., Rosli, M. M. & Fun, H.-K. (2010). *Acta Cryst.* E**66**, o1824–o1825. 10.1107/S1600536810023639 PMC3006730 21588031

[bb9] Hwu, J. R., Singha, R., Hong, S. C., Chang, Y. H., Das, A. R., Vliegen, I., De Clercq, E. & Neyts, J. (2008). *Antivir. Res.* **77**, 157–162. 10.1016/j.antiviral.2007.09.003 17977606

[bb10] Li, Y.-F., Wang, G.-F., He, P.-L., Huang, W.-G., Zhu, F. H., Gao, H.-Y., Luo, Y., Feng, C.-L., Shi, L.-P., Ren, Y.-D., Lu, W. & Zuo, J.-P. (2006). *J. Med. Chem.* **49**, 4790–4794. 10.1021/jm060330f 16854087

[bb11] Preston, P. N. (1974). *Chem. Rev.* **74**, 279–314.

[bb12] Ruiz, V. R., Corma, A. & Sabater, M. J. (2010). *Tetrahedron*, **66**, 730–735.

[bb13] Sasmal, P., Sasmal, S., Abbineni, C., Venkatesham, B., Rao, P. T., Roshaiah, M., Khanna, I., Sebastian, V. J., Suresh, J., Singh, M. P., Talwar, R., Shashikumar, D., Reddy, K. H., Frimurer, T. M., Rist, O., Elster, L. & Hogberg, T. (2011). *Med. Chem. Commun.* **2**, 385–389.

[bb14] Sheldrick, G. M. (2008). *Acta Cryst.* A**64**, 112–122. 10.1107/S0108767307043930 18156677

[bb15] Spek, A. L. (2009). *Acta Cryst.* D**65**, 148–155. 10.1107/S090744490804362X PMC2631630 19171970

[bb16] Wright, J. B. (1951). *Chem. Rev.* **48**, 387–541. 24541208

